# Rapid Forgetting Results From Competition Over Time Between Items in Visual Working Memory

**DOI:** 10.1037/xlm0000328

**Published:** 2016-09-26

**Authors:** Yoni Pertzov, Sanjay Manohar, Masud Husain

**Affiliations:** 1 Department of Psychology, The Hebrew University of Jerusalem; 2 Nuffield Department of Clinical Neurosciences, John Radcliffe Hospital, Oxford, United Kingdom, and Department of Experimental Psychology, University of Oxford

**Keywords:** forgetting, working memory, binding, attention, biased competition

## Abstract

Working memory is now established as a fundamental cognitive process across a range of species. Loss of information held in working memory has the potential to disrupt many aspects of cognitive function. However, despite its significance, the mechanisms underlying rapid forgetting remain unclear, with intense recent debate as to whether it is interference between stored items that leads to loss of information or simply temporal decay. Here we show that both factors are essential and interact in a highly specific manner. Although a single item can be maintained in memory with high fidelity, multiple items compete in working memory, progressively degrading each other’s representations as time passes. Specifically, interaction between items is associated with both worsening precision and increased reporting errors of object features over time. Importantly, during the period of maintenance, although items are no longer visible, maintenance resources can be selectively redeployed to protect the probability to recall the correct feature and the precision with which cued items can be recalled, as if it was the only item in memory. These findings reveal that the biased competition concept could be applied not only to perceptual processes but also to active maintenance of working memory representations over time.

Most of our memories last very briefly (Muter, 1980; Wixted, 2004). Rapid forgetting - apparent loss of information over just a few seconds - is particularly prominent with aging and is now recognized as a potential pathological marker for developing Alzheimer’s disease (Gagnon & Belleville, 2011). Even in young people, the ability to hold information in working memory (WM) over a few seconds correlates well with established tests of general intelligence (Conway, Kane, & Engle, 2003). In fact, WM is now considered to be a fundamental cognitive process across a range of species (Elmore et al., 2011; Kawai & Matsuzawa, 2000; Light et al., 2010; Wright et al., 2010).

The controversy about forgetting can be traced back more than a century to Thorndike’s law of disuse: “When a modifiable connection is not made between a situation and a response during a length of time, that connection’s strength is decreased” (Thorndike, 1913). The implication is that disuse—and therefore the passage of time—by itself produces forgetting. The effect of mere temporal decay on short-term forgetting gained prominence with Baddeley’s phonological loop model (Baddeley, Thomson, & Buchanan, 1975), which suggested that active rehearsal is needed to overcome time-related decay of memory. Evidence for the passage of time being the major factor in forgetting continues to be an important feature of several studies (Barrouillet & Camos, 2009; Barrouillet, De Paepe, & Langerock, 2012; Vergauwe, Barrouillet, & Camos, 2009).

On the other hand, not long after the “law of disuse” was formulated, a long debate was initiated with the claim that time itself is not the most important factor behind forgetting (Cason, 1924), with analogies made, for example, to the fact that time alone does not transform iron to rust (McGeoch, 1932). Thus, McGeoch (1932) suggested that the significant factors behind forgetting are “interpolated activities and changed stimulating conditions” rather than passage of time. Similar concepts are invoked today by researchers who strongly argue for a crucial role of interference in memory from distracting processes (Lewandowsky, Duncan, & Brown, 2004; Lewandowsky et al., 2009b; Oberauer & Lewandowsky, 2008). As yet, there is no resolution to this debate.

It is important to note that most previous studies that have examined this issue have used either verbal stimuli that require participants to remember strings of numbers, letters, or words or visual tasks that require them to detect a change in two successive presentations of an array (Baddeley, 2007; Brockmole, 2009; Della Sala, 2010; Melton, 1963; Posner & Konick, 1966). However, the fact that only two possible outcomes can be registered (correct or incorrect) somewhat constrains the amount of information that might be extracted from these tasks. An alternative type of paradigm, often called “delayed estimation task,” requires participants to reproduce a feature in memory on a continuous scale of report (Bays, Catalao, & Husain, 2009; Prinzmetal, Amiri, Allen, & Edwards, 1998; Wilken & Ma, 2004; Zhang & Luck, 2008), enabling the analysis of the distribution of error in recall. This method has been used successfully to challenge current views regarding capacity limits in visual WM (Bays et al., 2009; Bays & Husain, 2008; Wilken & Ma, 2004) and provides a more sensitive means to probe memory than traditional tasks (Zokaei, Burnett Heyes, Gorgoraptis, Budhdeo, & Husain, 2014).

Two recent studies have addressed forgetting using a delayed estimation task that enabled the analysis of the distribution of errors. Zhang and Luck (2009) asked participants to remember three patches of color or shapes. After a variable retention interval of up to 10 sec, participants were required to reproduce the correct feature out of a continuous scale. A mixture model analysis distinguished between random errors (presumably a result of a failure to access the target information at the time of test) and the precision of recall. Forgetting was found to reflect a lower probability of recalling the target, but crucially not in precision.

Another recent study used a similar approach but also manipulated the time between consecutive trials (Souza & Oberauer, 2015). This manipulation turned out to have strong impact on recall accuracy, supporting the “temporal distinctiveness” hypothesis (Brown, Neath, & Chater, 2007). According to this theory, time serves as a retrieval cue for a target event, and when events are crowded close together temporally, they are more difficult to retrieve. Mixture model analysis revealed that temporal distinctiveness affects the probability of correctly retrieving information from WM, but not its precision, somewhat in agreement with the finding of Zhang and Luck (2009). However, it is critical to note that both studies did not manipulate the number of items participants were required to remember.

The most direct way to study how additional items in memory influence forgetting is by comparing forgetting slopes when participants try to remember different memory loads. Such a strategy has been deployed previously in pioneering studies using verbal material (Melton, 1963; Posner & Konick, 1966). Those experiments concluded that interference between items held in memory plays a crucial role in their recall, with greater memory loads leading to greater attrition of recall over time. Thus, manipulating the number of items in the memory array as well as the retention duration is crucial for understanding how items interact in memory. However, previous studies have relied on binary report (correct/incorrect) and, to the best of our knowledge, combined manipulation of load and retention interval has not been examined for visual objects using the delayed estimation method.

Here we test memory for variable number of visual items over different durations to examine how interaction among items in memory contributes to rapid forgetting. First, we tested the fidelity of WM recall by using a delayed estimation task and subjected the results to a mixture model analysis. Using this technique, we show that a single item can be maintained in memory with high fidelity over the short term. However, if further items are added, they degrade each other’s representation as time passes: competing with each other in memory, just as one prominent theory suggests objects compete for visual processing resources when they are visible (Desimone & Duncan, 1995). Our findings reveal that for visual WM, this competition specifically results in increasing variability in recall as well as progressive loss of feature bindings—information that correctly holds together the component features that belong to particular objects.

Second, we compared our findings to a recent investigation of “retro-cuing”—cuing one item long after the memory array has been removed—to examine how forgetting is influenced by directing attention toward a single representation in memory (Pertzov, Bays, Joseph, & Husain, 2013). In that study, we used identical stimuli to the ones used in the current one and showed that the selected or attended memory representation is forgotten far more slowly than the other items in memory. Again, analogous to the concept of biased competition in visual attention (Desimone & Duncan, 1995), it seems that rapid forgetting could be prevented by biasing memory to a cued item and, importantly, simultaneously leading to faster forgetting of uncued items. We were able to compare the forgetting slopes for one and four items to the rate of forgetting slopes for the retro-cued item in our previous research to determine whether the retro-cued item is protected as if it was the only item in memory.

The results show that rapid forgetting involves an interaction between time and the number of items to be held in memory, with competition between stored objects leading to accelerated degradation of their representations. Furthermore, biasing memory resources to a specific item in memory can protect it from loss, with the same fidelity as if it was the only item in WM.

## Method

### Experimental Procedure

Ten neurologically normal participants (age range 19–35 years) participated after giving informed consent. All reported normal or corrected-to-normal visual acuity. Stimuli were presented at a viewing distance of 60 cm on a 21-inches cathode-ray tube monitor. Each memory array consisted of oriented bars (2° × 0.3° of visual angle) presented on a gray background on an imaginary circle (radius 4.4°) around fixation with equal interitem distances (center to center). The colors of the bars in each trial were randomly selected out of eight easily distinguishable colors. Bars within the same trials differed by at least 10° in orientation, which was otherwise random.

Each trial began with the presentation of a central fixation cross (white, 0.8° diameter) for 500 milliseconds (ms), followed by a memory array. Each of the participants performed 10 practice trials and between 11 and 15 blocks of 80 trials. Each block consisted of 20 trials for each of the four possible set sizes (1, 2, 4 and 6 bars), consisting of 5 trials for each delay duration (0.1, 1, 2 and 3 sec). At the end of each sequence, recall for one of the items was tested by displaying a “probe” bar of the same color with a random orientation. Subjects were instructed to rotate the probe using a response dial (PowerMate, Griffin Technology, Nashville, TN) to match the remembered orientation of the item of the same color in the sequence—henceforth termed the *target*. Note that we use the term *target* here simply to distinguish from other items, or nontargets, that were not probed.

### Analysis

**Figure 1 fig1:**
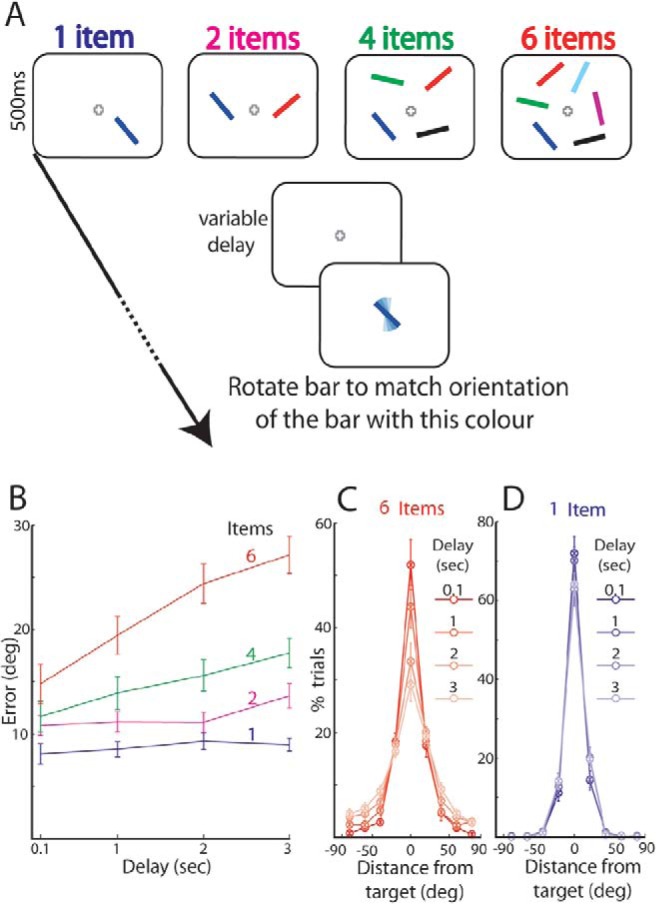
Forgetting with time as a function of number of items in memory. (A) Experimental design: 1, 2, 4, or 6 bars with different orientations and colors were presented for 500 ms. After a variable delay period, a probe item with the color of one of the items (in this example, blue) was presented, and subjects adjusted the orientation of the probe to match the remembered orientation of the item with the same color. (B) Mean error of recall for increasing set sizes and delays. (C) Distribution of responses for increasing delays, plotted with respect to target orientation (aligned at 0) when 6 items were presented. Different shades represent different delays. Note how the distribution of errors in recalling target orientation alters with delay. (D) Distribution of responses for one item did not alter with different delays. Error bars denote *SEM* across participants. deg = degrees; sec = seconds; ms = milliseconds. See the online article for the color version of this figure.

For each trial, a measure of raw error was obtained by calculating the angular deviation between the orientation reported by the subject and the orientation of the target item. These values were averaged separately for the different trial conditions and durations of delay. The raw error values for each participant and condition were divided into bins of 20° and presented as histograms in Figure 1C and 1D.[Fig-anchor fig1]

To quantify the contribution of different sources of error to overall errors, we applied a probabilistic mixture model introduced previously by Bays et al. (Bays et al., 2009; Bays, Wu, & Husain, 2011), which elaborated an earlier model by Zhang and Luck (2008).

**Figure 2 fig2:**
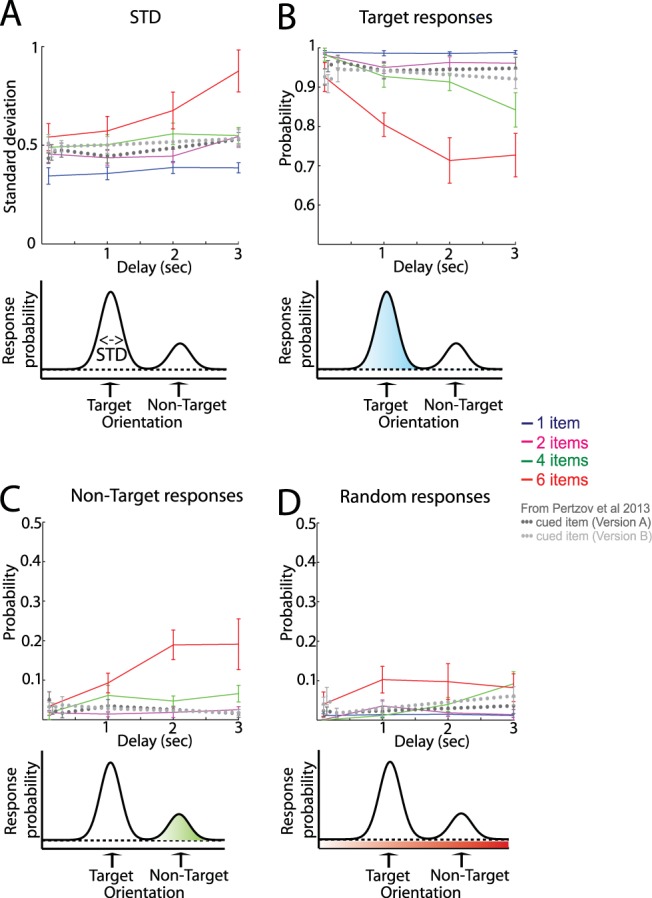
Results of probabilistic model of sources of error in responses. (A) Variability in recall of each item’s orientation is shown as the *SD* of the underlying error distribution. Participants’ responses were decomposed into 3 further separate components, illustrated by the colored regions in the illustrations: (B) a circular Gaussian distribution of responses centered on the orientation value of the target; (C) circular Gaussian distributions with the same width centered on each nontarget orientation value, corresponding to misbinding errors; and (D) and a uniform distribution, capturing random responses unrelated to any of the sample orientations. Different colors represent different number of items. For comparison, dotted lines in dark and light gray show model results of the single, retro-cued item in the two experiments reported in Pertzov, Bays, Joseph, & Husain, 2013, respectively. Error bars denote *SEM* across participants. STD = standard deviation parameter; sec = seconds. See the online article for the color version of this figure.

This model attributes the distribution of responses on the estimation task to a mixture of three components (illustrated in Figure 2), corresponding to reporting the target orientation (Figure 2B), mistakenly reporting one of the other (nontarget) orientations in the memory array (Figure 2C), and responding at random (Figure 2D). Orientations of all memory array items are recalled with a Gaussian variability. Mathematically, the model is described by the following equation:

$$p(\hat{\theta })=\alpha \phi_{k}(\hat{\theta }-\theta )+\beta \frac{1}{m}\sum_{i=1}^{m}\phi_{k}(\hat{\theta }-\varphi_{i})+\gamma \frac{1}{2\pi }$$

where θ is the true orientation of the target item, $\hat{\theta }$ is the orientation reported by the subject, and $\phi_{k}$ is the von Mises distribution (the circular analogue of the Gaussian) with mean zero and concentration parameter κ. The probability of reporting the correct target item is given by α. The probability of mistakenly reporting a nontarget item is given by β, and {$\varphi_{1},\;\varphi_{2,\;. . .\;}\varphi_{m}\}$ are the orientations of the *m* nontarget items. The probability of responding randomly is given by γ = 1 – α – β. Maximum likelihood estimates of the parameters α, β, γ, and κ were obtained separately for each subject, condition, and delay interval using an expectation maximization algorithm. Concentration parameter κ was converted to the more familiar standard deviation (Figure 2A) according to the method of Fisher (1995) (MATLAB code available at: http://www.paulbays.com/code/JV10/index.php). A 4 × 4 repeated-measures analysis of variance (ANOVA) with factors *set size* (1, 2, 4, and 6 items) × *delay duration* (0.1, 1, 2, and 3 sec) was conducted on the subjects’ errors and estimated parameters related to the three types of error. We performed a linear regression to calculate the slope of error across time for the different delay intervals for each participant.[Fig-anchor fig2]

## Results

### Temporal Delay and Number of Items Both Modulate WM Precision

Participants were briefly presented with randomly oriented colored bars and, after a variable delay, were asked to reproduce from memory the orientation of one of the bars, specified by its color (Figure 1A). The number of stimuli presented (set size) varied among 1, 2, 4, and 6 in a randomized, interleaved manner.

The error with which subjects recalled an item’s orientation (Figure 1B) increased with delay duration, *F*(3, 27) = 65.76, *p* < .001, as well as with set size, *F*(3, 27) = 56.23, *p* < .001. It is important to note that the interaction between these factors was also significant: the gradient of the error function showed a clear increase in the rate of forgetting with increasing set size (Figure 1B; *F*(9, 81) = 19.02, *p* < .001).

With a large number of items held in memory, longer delays led to a decrease in the number of very precise responses (e.g., six items, responses with <10 degrees of error, effect of delay: *F*(3, 36) = 6.15, *p* = .002) and a corresponding increase in the number of trials with large errors (e.g., six items, responses between 30 and 50 degrees of error, effect of delay: *F*(3, 36) = 5.43, *p* = .004). Figure 1C shows for a set size of 6 how the distribution of responses, aligned to the true target orientation, alters with increasing delay durations (marked in different shades). By contrast, when only a single item had to be remembered, delay duration had little influence on the distribution of responses (Figure 1D; one item, responses with <10 degrees of error, effect of delay: *F*(3, 36) = 1.32, *p* = .3; responses between 30 and 50 degrees of error: *F*(3, 36) = 0.21, *p* = .9).

The increased errors with larger delay durations and set sizes might be attributable to three different factors: noisier representation of the target (or probed) object; higher probability of reporting nontarget orientations (indicating erroneous binding—or misbinding—of the target color with the orientation of another item that appeared in the array); or finally an increase in random responses, guessing unrelated to any of the orientations shown in the array.

### Decomposing Errors into Three Sources

To investigate the different sources of error, we applied to the data a probabilistic mixture model that assumes these three potential sources of error (Bays et al., 2009; Fougnie, Asplund, & Marois, 2010). Figure 2 presents the results of the mixture model analysis. The standard deviation parameter (STD), which is proportional to the width of the underlying memory distribution (Figure 2A), was significantly modulated by both delay, *F*(3, 27) = 15.48, *p* < .001, and set size, *F*(3, 27) = 19.00, *p* < .001. This is consistent with the view that higher memory load as well as longer delays lead to broader distribution of responses. The interaction was also significant, *F*(9, 81) = 3.70, *p* < .001, consistent with a more stable precision for a single memorized item but worsening variability with time at larger set sizes.

Figure 2B shows the probability that the response was drawn from the distribution centered on the correct target orientation. Again, both delay, *F*(3, 27) = 9.41, *p* < .001 and set size, *F*(3, 27) = 31.70, *p* < .001, significantly influenced the likelihood of participants responding with the correct target orientation, with the interaction between these factors being significant, *F*(9, 81) = 3.18, *p* = .002. Thus, longer delays as well as larger set sizes decreased the probability that a response reflected noisy recall of target orientation as opposed to a nontarget or random response.

What about misbinding target color with a nontarget’s orientation? We can examine this issue by assessing the probability that the response is centered on the orientation of one of the nontarget items (items presented in the original array but not probed). Such misbinding was found to be a key ingredient, with this type of error increasing with set size and delay duration (main effect of set size: *F*(3, 27) = 26.55, *p* < .001, delay duration: *F*(3, 27) = 3.39, *p* = .032), with a significant interaction between these factors, *F*(9, 81) = 2.53, *p* = .013.

Increasing set size and delay duration also led to an increase in uniformly distributed or random responses (i.e., centered neither on target nor nontarget orientations; main effect of set size: *F*(3, 27) = 6.24, *p* = .002, delay duration: *F*(3, 27) = 3.73, *p* = .023), but with no significant interaction in this case, *F*(9, 81) = 1.21, *p* > .3. Overall, the more prominent type of error was a systematic biasing of responses to the orientation of nontargets—misbinding responses. For example, for a memory load of six items and >1 sec delay, the probability of responding with the orientation of a nontarget item was twice as high as responding at random (19% vs. 9%; Figure 2C and 2D).

### Biasing Competition in WM

WM is not simply a passive storage buffer, but rather a system capable of processing and manipulating (“working”) with stored representations (Baddeley, 1992, 2007). We have previously investigated how high-level goals change the temporal dynamics of memory representations. In analogy to the biased-competition account of visual processing, we investigated whether forgetting slopes could be biased by top-down processes using a procedure called retro-cuing (e.g., Griffin & Nobre, 2003). In this design, a cue is presented well after sample stimuli have been extinguished, typically leading to enhanced detection of a change in later test stimuli (e.g., Gazzaley & Nobre, 2012; Griffin & Nobre, 2003; Kuo, Rao, Lepsien, & Nobre, 2009; Lepsien & Nobre, 2006). In a previous study, we combined retro-cuing (of 70% validity) with the same delayed estimation task we used here, across variable delays, to study whether forgetting slopes could be biased by retro-cueing. In our previous investigation, we used an identical setup (stimuli dimension, screen, report methods etc.) to compare the forgetting slopes of retro-cued items to the forgetting slopes of items without cueing. We can now ask whether a retro-cued item is forgotten at the same rate as if it was the only item displayed and held in WM.

**Figure 3 fig3:**
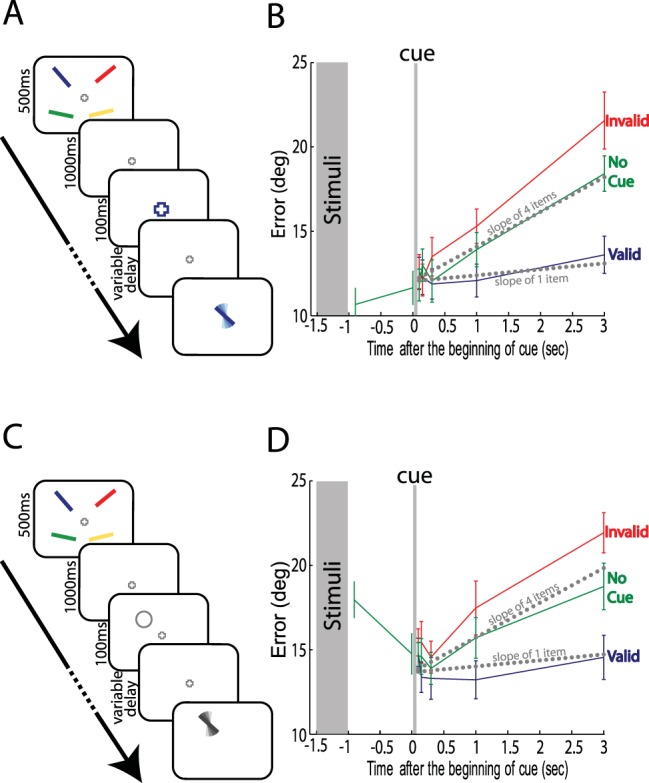
Retro-cue experiments: biasing within WM. (A) Previous color probe, retro-cue experiment. Participants saw 4 bars, each with a different orientation and color, for 500 ms. In most trials, after 1 sec of blank display, the fixation color changed to match one of the preceding bars (enlargement of fixation point is only for presentation purposes). This signaled the most probable (70%) item to be probed. In a proportion of trials no cue was presented. In all trials, a probe item with the color of one of the items (in this example, blue) was presented after a variable delay, and subjects adjusted the orientation of the probe to match the remembered orientation of the item with the same color. (B) Error in recall over time for the three different conditions: blue = valid, cue matches the probe; red = invalid, cue does not match the probe; green = no cue, no cue was presented. Gray dotted lines represent the predicted performance using the slopes calculated from 1 and 4 item conditions in the current experiment. (C) Previous location probe, retro-cue experiment. Similar to (A), but the cue was a gray ring displayed at the location of the probable target. The probe bar was presented in a neutral color at the location of one of the memory items (70% at cued location). (D) Results of probe-by-location experiment. Error bars denote *SEM* across participants. ms = milliseconds; sec = seconds; deg = degrees. See the online article for the color version of this figure.

Figure 3A and 3C, illustrates the experimental design we used in two previous tasks (Pertzov et al., 2013), based on probing memory either by color or by location. In 70% of trials a retro-cue was presented 1 sec after the sample stimuli had been extinguished. When a cue was presented, it corresponded to the item that was subsequently probed (valid condition) on 70% of trials and one of the other items (invalid condition) on the rest. The probe in no-cue trials was presented at various delays after the stimuli presentation. These delays matched the delays of the cued trials with the addition of two further time points 0.1 and 1 sec after the stimuli was extinguished (for more detailed experimental settings, please see Pertzov et al., 2013).[Fig-anchor fig3]

**Figure 4 fig4:**
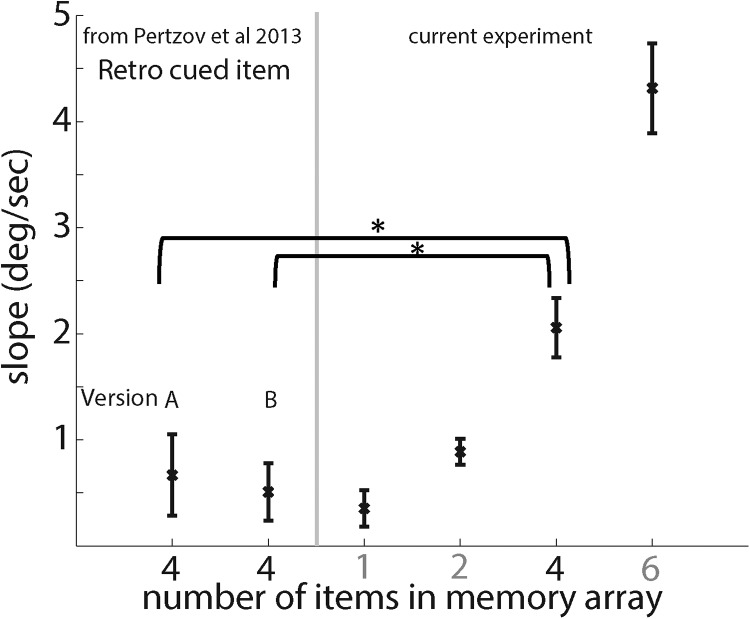
Comparison of forgetting slopes. The right side of the figure shows slopes of linear regression between averaged error and delay interval for the different number of items in the current experiment. For comparison, we also calculated the forgetting slopes of the retro-cued items from our previous experiments (Pertzov et al., 2013) shown in Figure 3. These are shown on the left of the graph. Note that the retro-cued items were forgotten similarly to the one item condition and significantly more slowly than the 4-items slope although 4 items were displayed. Error bars denote *SEM* across participants. deg = degrees; sec = seconds. * *p* < .05.

In both experiments, the fidelity with which the cued item was recalled was relatively stable across time (Figure 3 blue line; mean slope of 0.47 deg/sec for the probe-by-color task and 0.22 deg/sec for the probe-by-location task). These slopes were not significantly different from zero (Figure 4; *t*(11) < 1.5; *p* > .16). Crucially, they were comparable to the slope of 1 memorized item in the current experiment (mean slope of 0.35 deg/sec; dotted gray line in Figure 3; *t*(20)s < 0.7; *p*s >0.5) as illustrated in Figure 4. Importantly, although four items were displayed before the cue, the retro-cued forgetting slopes were significantly lower than the slopes of four items in our experiment (*t*(20)s > 2.6; *p*s < 0.015).[Fig-anchor fig4]

The fact that the temporal gradient of the cued representation’s fidelity is similar to that observed when only one item is held in memory (although four items were actually displayed) suggests that maintenance resources can indeed be dynamically reallocated according to new task goals and thereby bias competition-based forgetting toward a selected memory representation.

### Model Effects: Deployment of Resources to a Cued Item Leads to Stable *SD* and Misbinding

Next we applied the mixture model analysis to the responses gathered from the retro-cue tasks and plotted it on top of the mixture model results of the current experiment (gray dotted lines in Figure 2). Consistent with the raw error analysis (Figure 3), model parameters of cued items were stable across time, just as if one item had been presented in the to-be-remembered array (compare slopes of gray dotted lines to blue lines in Figure 2). Note that comparisons across experiments are meaningful only by examining gradients of performance over time because the absolute values of the retro-cue results are determined by the encoding stage (in which four items were always presented) and the time that passed before the cue was extinguished (1.1 sec). We conclude from this analysis that selective deployment of maintenance resources can provide protection from the progressive deterioration in precision as well as misbinding that results from holding multiple items in memory (Figure 2B).

## Discussion

We studied the fidelity with which visual items are retained in WM, manipulating both set size and delay duration and using precision of recall as an index. Studying the forgetting slopes enabled us to assess the dynamics of maintenance in WM representations without confounds related to visual processing or encoding of the memory array. First, we found that greater temporal delays lead to forgetting, but crucially only when multiple items must be remembered (Figure 1). This is inconsistent with the hypothesis that time alone determines forgetting—at least over the intervals we have studied—which predicts a similar rate of forgetting for different numbers of objects in memory. The fact that forgetting slopes are steeper for larger numbers of items in memory is not trivial: it provides strong evidence for interaction among different items held in memory, a concept that would not be an obvious prediction of pure “slot” models of WM (e.g., Luck & Vogel, 1997) in which each object is assumed to be stored independently, without cross-talk among those representations. However, note that the slot model could be consistent with our results by adding a limited supporting process that is shared among all items, such as a serial rehearsal or reactivation process.

The results presented here also challenge any time-invariant role of interference in forgetting because such accounts by definition predict zero forgetting slopes. Thus, short-term forgetting is mediated by mutual competition among memorized representations that leads to worse performance over time. However, time alone is not sufficient: rapid forgetting requires both competition and time. The competition between items is reflected as both increasing variability and increasing probability in reporting the wrong item in memory (Figure 2).

Our conclusion that forgetting is related to time-dependent interference between items in memory is in agreement with two pioneering studies conducted more than 50 years ago using verbal stimuli and binary report (correct/incorrect response). Those investigations showed that either additional (Melton, 1963) or more similar letters (Posner & Konick, 1966) to be remembered lead to steeper forgetting curves across filled retention intervals. Posner and Konick concluded colorfully that forgetting was akin to an “acid bath,” with greater degradation occurring with more retained items (analogous to the concentration of acid) and increasing maintenance time (within the acid). However, to the best of our knowledge the same conclusions have not been demonstrated for visual memoranda using a delayed estimation task as used here. Moreover, the modeling we used shows far more directly that the competition is one of interference between retained features belonging to different objects, leading to misbinding reports. Previous studies using verbal stimuli did not do this.

In two previous experiments (Pertzov et al., 2013) we found that rapid forgetting is not “compulsory.” Subjects were able to bias interitem competition in favor of one representation—retro-cued either by object color or location—and protect it from degradation (Figure 3; Pertzov et al., 2013). Strikingly, the forgetting slope of a retro-cued item in a four-item array was comparable to the rate of forgetting when only a single item was held in memory (comparison of data from the current and previous experiment; Figures 3 and 4). Thus, top-down processes can bias resources dedicated to the maintenance of each memory representation and counteract the competition induced by other memory representations. Specifically, they maintain the fidelity of memory for the prioritized object, including the associations or bindings among its different features, as if it was the only object in memory.

What is the nature of information that is being degraded over time in our experiments? Is it sensory or categorical? Although the longest duration used (3 secs) is too long for iconic memory, recent work has proposed that a high capacity but fragile visual short-term store, different from visual WM, might operate over such a time interval (Pinto, Sligte, Shapiro, & Lamme, 2013; Vandenbroucke, Sligte, & Lamme, 2011). Such a system may be very much a sensory memory because it is erased if similar objects are presented at the same locations (Pinto et al., 2013). The existence of such a longer lasting but fragile store is highly controversial, with some arguing that the effects emerge only after a long period of practice (Matsukura & Hollingworth, 2011).

Although it is possible that the competition we have observed among items in memory occur at this level, we consider this unlikely for three reasons. First, we are not aware of any previous data that show that this fragile store is characterized by competition that leads specifically to misbinding of features belonging to different items. By contrast, active binding of visual features is considered to be a key part of WM (Treisman & Zhang, 2006; Wheeler & Treisman, 2002). Second, the fragile short-term memory (STM) system is typically revealed if a retro-cue is presented before probing. In the main experiment used here, we did not use such a cue. Rather, we used only a probe that is most likely to diminish the contribution of such “fragile memory” traces. Finally, it has been shown that prolonged practice might be required to demonstrate the fragile memory effect (Matsukura & Hollingworth, 2011). In our study, a long period of practice was not made available to participants. Nevertheless, it remains a possibility that the interference we have observed over time might be related to competition within such a sensory memory store.

In visual perception, competition among items in the scene is considered to be crucial (Desimone & Duncan, 1995). More recently, others have suggested that such competition might also operate in WM (Shapiro & Miller, 2011), possibly via a two-dimensional map architecture (Franconeri, Alvarez, & Cavanagh, 2013) in which nearby items inhibit each other (Emrich & Ferber, 2012; Kiyonaga & Egner, 2015). Our data are consistent with this approach: items become closer to each other when more of them are displayed and therefore are expected to interfere more with each other. However, note that we cannot make any conclusions regarding the exact mechanism of interitem interference that leads to our results. In fact, an alternative interference account that does not rely on spatial-based interference but rather on time-sharing might also provide a plausible account (see below). In any event, the novel contribution of our study is that it provides compelling evidence that interitem competition, resembling that observed in visual processing, also occurs during maintenance of objects in visual WM. Such maintenance competition could only be revealed by analyzing the forgetting slopes when a variable number of items are retained in memory. As far as we are aware, this is the first report that convincingly shows that more items held in visual WM lead to faster forgetting slopes.

A second important result of this study is the modeling used to examine the nature of errors made over time. Maintenance competition appears to lead to mutual degradation of item representations in a specific manner. The analysis shows that this is via an increase in probability of misbinding one object’s features with another’s, relating directly to the concept of attention as a binding mechanism within visual processing (Treisman & Gelade, 1980). Critically, our experimental design controls well for visual processing because the forgetting slopes are not sensitive to the initial stages of visual perception (captured in the shortest delay of 100 ms; reflected in the intercepts of the forgetting functions rather than their gradients). Therefore, maintenance of items in WM seems to be supported by a mechanism that functions similarly to perceptual attention, but even when visual information is no longer present (Chun, 2011; Wheeler & Treisman, 2002), a view consistent with the new taxonomical definitions of “internal” or “reflective” attention (Chun, Golomb, & Turk-Browne, 2011; Chun & Johnson, 2011).

In contrast to the findings we report, a study using a probabilistic model of errors on a recall task concluded that over time there is an increase in the probability of responding randomly, but no increase in recall variability (Zhang & Luck, 2009). The authors of that report concluded that forgetting is effectively the result of “sudden death” (complete erasure) of memory representations (Zhang & Luck, 2009). However, it is critical to note that they tested only small arrays of three items. Here we used a wider range of set sizes and found evidence for progressive changes in recall variability over time (i.e., gradual decay—not only sudden death—of memory representations). We note also that the sudden death observed by Zhang and Luck occurred using a design with a much longer delay than the longest interval used here (10 vs. 3 sec). It is possible that such errors might gain greater prominence when the retention interval is extended to longer delays. In addition, the previous analysis assumed that any response not centered on the target orientation was a random guess; misbinding errors were not modeled. The recall task requires not only that a subject correctly remember the target feature of the items in the memory array (i.e., orientation in the present study) but also that each target feature is correctly matched (“bound”) with the corresponding probe feature (i.e., color; Bays et al., 2011; Pertzov & Husain, 2014).

Nontarget stimuli have been found to act as a strong attractor on the recalled appearance of an accompanying target stimulus (Bays, 2016; Huang & Sekuler, 2010). Our analysis also shows that of the responses not centered on the target orientation at larger set sizes, the majority are due to incorrectly reporting of a nontarget item rather than random responding. Thus, misreporting features that belong to different objects stored in WM (Bays et al., 2011; Ma, Husain, & Bays, 2014) plays a key role in rapid forgetting. In contrast, even the highest frequencies of random responding observed in the present study (∼10% in six-item arrays) are less than that predicted by the complete erasure of one item (16.7%). Thus, using a task with a continuous response permitted us to go beyond the results of previous seminal studies that showed that more complex visual stimuli are forgotten faster but are based on a binary measure of recall (Phillips, 1974).

Here we specifically found that increases in the number of items to be held in memory also increases the rate of forgetting, with forgetting manifests as a gradual decline in recall fidelity, caused by increases in both recall variability and misbinding of visual features over time. Such a view of forgetting is consistent, at least in part, with the concept of WM as active binding of visual features (Treisman & Zhang, 2006; Wheeler & Treisman, 2002) and extends findings that report the strong effect of delay interval on object-location binding across a few seconds (Pertzov, Dong, Peich, & Husain, 2012) and days (Lew, Pashler, & Vul, 2016).

What is the maintenance resource that items in memory compete for? A recent study presented data that show that although arrays of letters are not forgotten over short intervals, unconventional characters that are hard to name are (Ricker & Cowan, 2010). The authors hypothesized that their results could be explained as a combination of time-based forgetting and refreshing processes that are hampered in the unconventional characters condition. One type of refreshing process might be covert verbal rehearsal (Baddeley, 2007), which was found to counteract forgetting in a delayed estimation task when only one item had to be retained in memory (Donkin, Nosofsky, Gold, & Shiffrin, 2015). However, in our case, any crude verbal coding is highly unlikely to account for the levels of accuracy (errors <15 degrees of orientation) reported here. The maintenance resource items competed for in our type of experiment could instead be attentional refreshing (Barrouillet, Bernardin, & Camos, 2004), “covert visuospatial rehearsal” (Baddeley, 2007), or visual imagery (Baddeley, 2007). When more items are maintained in memory, the refreshing cycle would be longer, leading to a higher rate of forgetting reflected as loss of accessibility and precision. Indeed, a recent study has shown that items that were taken out of the focus of attention lead to less precise reports compared with items that reside in the focus of attention (LaRocque et al., 2015).

The time-based resource-sharing model (TBRS) provides one possible model of interitem competition. The TBRS model proposes a sequential and time-based sharing of the internal attention resource (Barrouillet et al., 2004; Barrouillet, Bernardin, Portrat, Vergauwe, & Camos, 2007) similar to the way a computer’s dynamic RAM is refreshed. To the best of our knowledge, TBRS models have not hitherto been discussed in the context of competition among multiple items. However, this model—among others—would be expected to generate faster forgetting slopes when the maintenance resources have to be shared among more items that reside simultaneously in memory. This view is supported by a recent study that reported that the time needed to refresh information in WM increases with the number of retained items (Vergauwe, Camos, & Barrouillet, 2014).

The processes underlying “attentional refreshing” or “visual imagery” are currently only vaguely defined (Baddeley, 2007). In this context, we regard the options discussed here as specific probable manifestations of the more general concept of maintenance resources. It is important to note that our findings help to characterize and constrain this resource. They suggest that when more items are maintained in memory, they share—and compete for—a limited pool of maintenance resources over time. Importantly, resources can be rapidly reallocated to a selected representation within WM and protect it from dissolving, as if it is the sole item in memory (Pertzov et al., 2013). Indeed, a recent imaging study (Lewis-Peacock & Norman, 2014) used pattern classification of functional magnetic resonance imaging data to show that switching between two representations in WM often leads to increased competition that results in strengthening of the winning memory and, crucially, simultaneous weakening of competing memories.

Finally, the findings presented here also have implications for everyday vision. Experiments using naturalistic tasks and free-viewing conditions suggest that participants store only very little task-related information because they tend to make eye movements to obtain the required information just before the moment they need it, leading to very brief retention intervals (Ballard, Hayhoe, & Pelz, 1995; Hayhoe, Bensinger, & Ballard, 1998). Our results might provide a rationale for such behavior: because retention of multiple objects leads to mutual degradation of their representations over time, it is most efficient to maintain a small number of items for short durations.

In conclusion, rapid forgetting occurs because items in WM compete and degrade each other’s representations as time passes. This competition manifests, in part, as decreased precision and failures in the binding of features that belong to objects held in memory. Maintenance resources can be dynamically reallocated to protect a selected item from competition and hold it with the same fidelity as a single retained item.
